# Full recovery of a 13-year-old boy with pediatric Ramsay Hunt syndrome using a shorter course of aciclovir and steroid at lower doses: a case report

**DOI:** 10.1186/1752-1947-5-376

**Published:** 2011-08-15

**Authors:** Gwinyai Masukume, Sheenah Chibwowa, Mbongeni Ndlovu

**Affiliations:** 1 Department of Medicine, Mpilo Central Hospital, Bulawayo, Zimbabwe

## Abstract

**Introduction:**

Reports on children with Ramsay Hunt syndrome are limited in the literature, resulting in uncertainty regarding the clinical manifestations and outcome of this syndrome. Treatment for Ramsay Hunt syndrome is usually with antivirals, although there is no evidence for beneficial effect on the outcome of Ramsay Hunt syndrome in adults (insufficient data on children exists). Here, we report a case of Ramsay Hunt syndrome occurring in a child who inadvertently received a lower dose of aciclovir and steroid administered for shorter than is usual. Our patient made a full recovery.

**Case presentation:**

A 13-year-old African boy presented to our out-patients department with an inability to move the right side of his face for one week. He had previously been seen by the doctor on call, who prescribed aciclovir 200 mg three times per day and prednisone 20 mg once daily, both orally for five days, with a working diagnosis of Bell's palsy. After commencement of aciclovir-prednisone, while at home, our patient had headache, malaise, altered taste, vomiting after feeds, a ringing sound in his right ear as well as earache and ear itchiness. Additionally, he developed numerous fluid-filled pimples on his right ear. On presentation, a physical examination revealed a right-sided lower motor neuron facial nerve palsy and a healing rash on the right pinna. On direct questioning, our patient admitted having had chicken pox about three months previously. Based on the history and physical examination, Ramsay Hunt syndrome was diagnosed. Our patient was lost to follow-up until 11 months after the onset of illness; at this time, his facial nerve function was normal.

**Conclusions:**

This case report documents the clinical manifestations and outcome of pediatric Ramsay Hunt syndrome; a condition with few case reports in the literature. In addition, our patient made a full recovery despite inadvertently receiving a lower dose of aciclovir and steroid administered for shorter than is usual.

## Introduction

Ramsay Hunt syndrome (RHS) type 2 is defined as peripheral facial paralysis accompanied by a vesicular rash on the ear (herpes zoster oticus) or in the mouth [1]. The syndrome is named for James Ramsay Hunt [2] (1874 to 1937), an American neurologist, who performed research on the entity that now bears his name [3]. It is caused by reactivation of the varicella zoster virus, which lies dormant in ganglia after usually having produced chicken pox during primary infection [4]. In children, the eruption of vesicles tends to be delayed [5].

Compared with adults, RHS is less frequent and less severe in children; however, its clinical manifestations and outcome are uncertain, as reports on children are limited in the literature [1]. Treatment for RHS is usually with antivirals, although there is no evidence for beneficial effect on the outcome of RHS in adults [1]. Regardless, lack of evidence does not necessarily mean antivirals are ineffective in RHS. We report a case of RHS occurring in a child.

## Case presentation

A 13-year-old African boy, in the company of his father, presented to the out-patients department at our facility with an inability to move the right side of his face for one week. Our patient's history was that he was well until 13 days prior to this presentation, when he developed a sore throat that resolved after two days. Two days after the sore throat resolved, during supper, it was noted that he would rest his head on his hands at the table, unlike his usual self. The next day, our patient woke up and reported that his face was feeling 'funny'. Later during that day, the father observed that his son's face was skewed and that he could no longer pronounce words properly.

Our teenage patient was taken to a medical facility where a course of amoxicillin was prescribed, and he was then referred to our hospital where aciclovir-prednisone was prescribed by the doctor on call (aciclovir 200 mg three times per day and prednisone 20 mg once daily, both orally for five days); the medication was commenced within two days of prescription. According to our patient's hospital records, the working diagnosis was Bell's palsy.

After commencement of aciclovir-prednisone, while at home, our patient had headache, malaise, altered taste, vomiting after food, a ringing sound in his right ear as well as earache and ear itchiness. Additionally, he developed numerous fluid-filled pimples on his right ear, prompting him to seek further medical care several days later when the pimples were already starting to heal (Table 1).

**Table 1:** Chronology of events from onset of illness

Day	Event(s)
1	Sore throat
2	Sore throat
3	-
4	-
5	Resting head on hands at supper table
6	Face feeling 'funny', face skewed, inability to pronounce words properly, inability to move right side of face, commences amoxicillin
7	-
8	Commences aciclovir, headache, blurred vision, (takes aspirin)
9	Commences prednisone, malaise, ringing sound right ear, vomiting after feeds, (takes aspirin)
10	Fluid-filled pimples on right ear, earache and ear itchiness, altered taste
11	Malaise, vomiting after feeds
12	Fluid-filled pimples beginning to 'dry', vomiting after feeds
13	Nausea, no longer vomiting, headache stops, ear ache decreasing
14	Day of presentation

On presentation, he denied headache, vomiting, earache, ever having a hot body or impaired hearing, but he admitted to feeling nauseous and having occasional itchiness of the right ear. He had never been admitted to the hospital for any reason previously, and his growth and development were normal according to his father. His parents and sibling currently have no known health problems.

**Figure 1:**
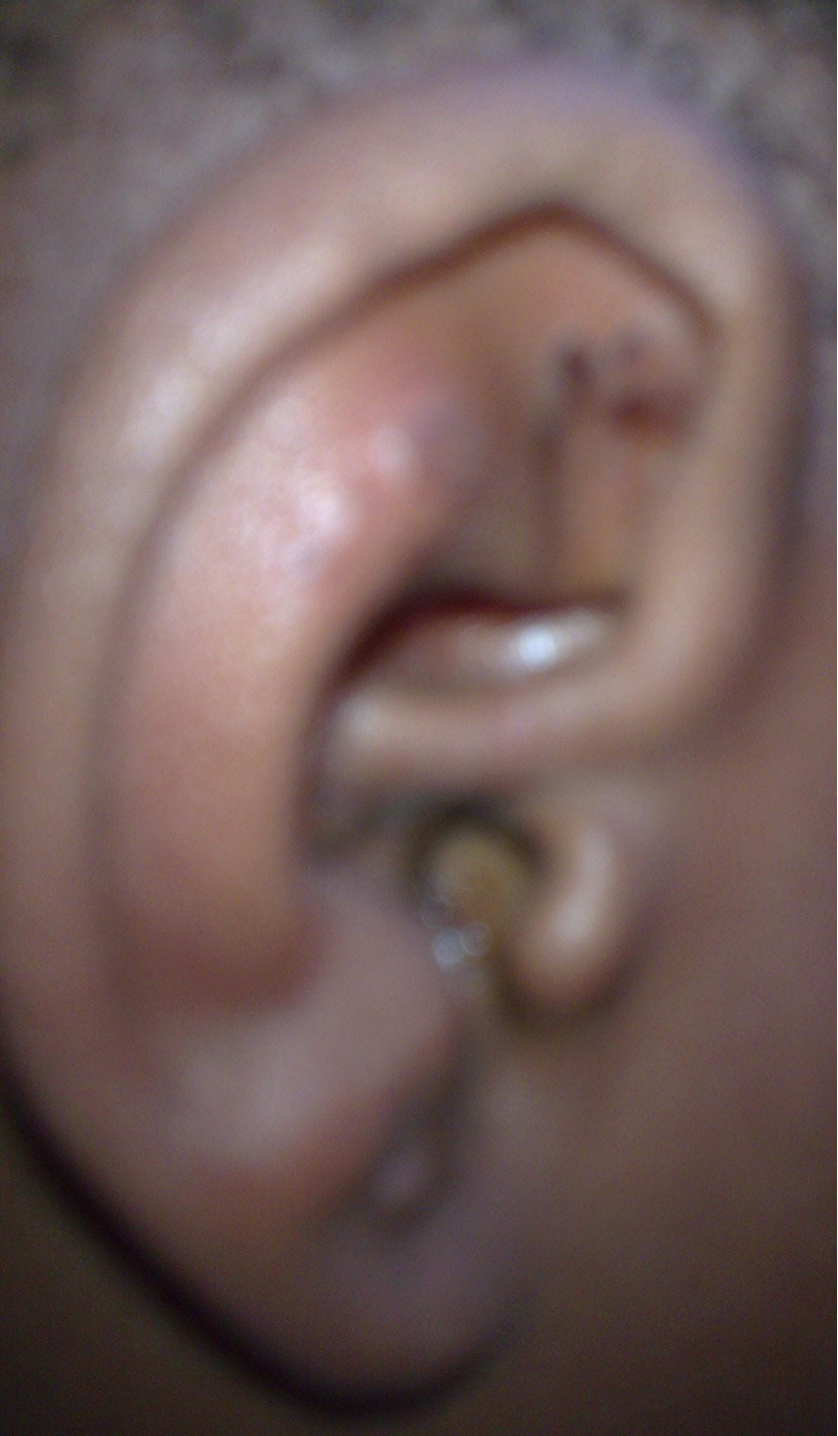
**Healing rash on the right pinna**. Note wax at the entrance of the external auditory meatus. Skin rashes are difficult to visualize on pigmented skin [12].

**Figure 2:**
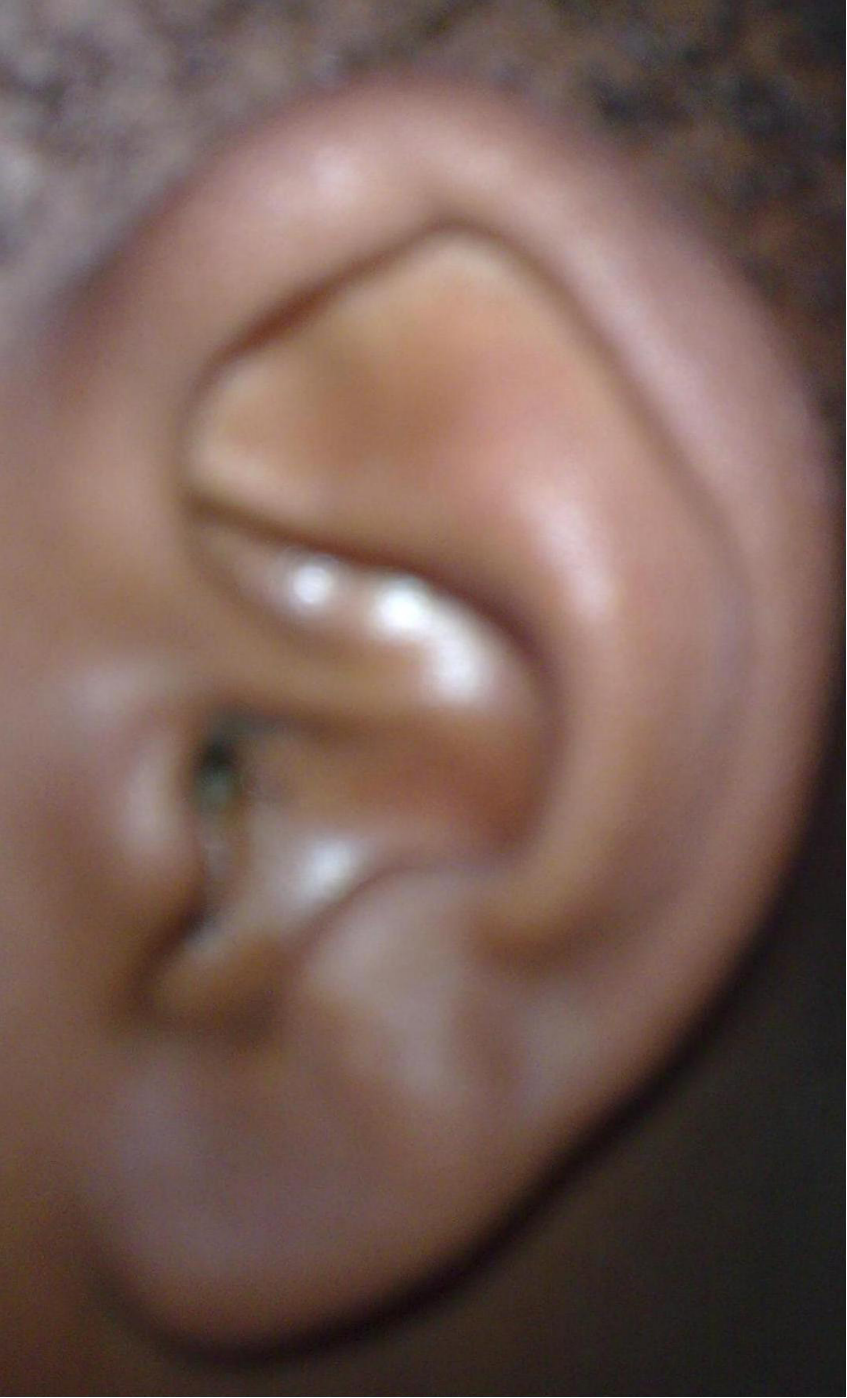
**The normal left pinna for comparison**.

Our patient weighed 36 kg. A physical examination revealed a right-sided lower motor neuron facial nerve palsy, healing rash on the right pinna (Figure 1; see also the normal left pinna for comparison in Figure 2), and loss of taste on approximately the right anterior half of the tongue. His facial nerve paralysis was grade IV (moderately severe dysfunction), using the House-Brackmann facial nerve grading system (ranging from I to VI, with I indicating normal function and VI indicating total paralysis). The rest of the examination was unremarkable (otoscopy was not performed). On direct questioning, our patient admitted having had chicken pox about three months previously; he had no prior vaccination against varicella zoster virus. Based on the history and physical examination, Ramsay Hunt syndrome was diagnosed. The diagnosis of Ramsay Hunt syndrome was explained to our patient and his father, advice on eye care was given and a referral for physiotherapy was made.

Our patient was lost to follow-up until 11 months after the onset of illness; at this time, his facial nerve function was normal (House-Brackmann grade I). An otoscopic examination was unremarkable. Our patient had apparently made a full recovery about one month from the beginning of sickness and had adhered to the suggested eye care and physiotherapy.

## Discussion

History taking and physical examination remain largely the basis of diagnosing RHS [6]. As the diagnosis of RHS was preceded by a sore throat, only becoming apparent after the eruption of ear vesicles on a background of peripheral facial paralysis, it was not unusual for our patient to have received an antibiotic course in primary care [7].

The symptoms of tinnitus, nausea and vomiting reported by our patient may be attributed to bystander involvement of the vestibulocochlear nerve [6], which traverses in close proximity to the facial nerve (affected in RHS) within the bony facial canal. Headache, nausea and at times vomiting are recognized common side effects of treatment with aciclovir [8]; this drug could have caused the aforementioned symptoms in our patient even though a lower dose for shorter than usual was used (adult dose, 800 mg orally five times per day for seven to 10 days) [8].

Self-medication by patients with aspirin is not uncommon [9]; being aware of this fact may prove useful. Herpes zoster complications appear more common in immunocompetent children [10] as our patient seemed to be. Childhood immunization against varicella zoster virus may prevent RHS, although there is concern the burden of disease may be shifted to adults [11].

Our patient may simply have had a spontaneous recovery independent of medication or may have recovered from aciclovir alone or steroid alone. The findings in adults from the Cochrane database review that we cite may not necessarily apply in the pediatric population. We cited the review in part to highlight that there is insufficient data on the pediatric population.

## Conclusions

This case report documents the clinical manifestations and outcome of pediatric Ramsay Hunt syndrome; a condition with few case reports in the literature. In addition, our patient made a full recovery despite inadvertently receiving a lower dose of aciclovir and steroid administered for a shorter period than is usual.

## Consent

Written informed consent was obtained from the patient's next-of-kin for publication of this case report and any accompanying images. A copy of the written consent is available for review by the Editor-in-Chief of this journal.

## Competing interests

The authors declare that they have no competing interests.

## Authors' contributions

GM, SC and MN contributed to the writing and editing of this article and approved the final version.
